# Awareness on mosquito borne diseases among urban & rural population in Northern Gujarat

**DOI:** 10.6026/97320630018640

**Published:** 2022-07-31

**Authors:** B Mahalakshmi, N Sivasubramanian, Payal Vaghela, Patel Divyankakumari Navinbhai, Patel hetal vasanthbhai, G Ramalakshmi, Gurav Priyanka Rajeshbhai, KJ Shaijo

**Affiliations:** 1 Nootan College of Nursing, Sankalchand Patel University, Visnagar, Gujarat - 384315, India; 2 Department of Community health Nursing, College of Nursing, S.G.R.R University, Dehradun, Uttarkhand - 248001, India

**Keywords:** Awareness, Precaution, Comparison, Mosquito Borne Diseases

## Abstract

It is of interest to examine the awareness and precautions of urban and rural residents on a number of mosquito-borne diseases (MBD). A sample of 300 adult people (Rural 150+Urban 150) was selected from Mahesana district of North Gujarat. Majority of samples (47.3 %) had average, 16% poor and 36.7% had good level of awareness in urban areas. In rural areas majority of the samples (40.67%) had average, 28%poor and 31.33% had good level of awareness. (67.3%) urban population were using mosquito repellent liquids and creams and (68.6%) of rural population were using mosquito net. Data shows that both urban and rural populations have moderate awareness on Mosquito Borne Diseases and majority is taking precaution towards these conditions. Data also revealed that there is no significant difference between urban & rural population's precaution measures against Mosquito borne diseases.

## Background:

Mosquito-borne infections account for over 17% of all infectious diseases and kill over 700,000 people each year. The most vulnerable population to malaria is children under the age of five [1]. Other viral infections spread by vectors include Chikungunya fever, Zika virus fever, yellow fever, and Japanese encephalitis. (All transmitted by mosquitoes) [2]. Insecticides sprayed in residences or applied to bed nets, and in the case of dengue vectors, reduction of larval breeding sites or larviciding with insecticides thereof, are currently being used to combat the enormous burden placed on populations worldwide by mosquito-borne diseases, most notably malaria and dengue [3]. The potential for mosquito and/or insecticide exposure around the home, and the access to these services by individuals of different socioeconomic categories is known [4]. In 2019, chikungunya was most prevalent in Asia and the Americas [5]. Zika virus can be sexually transmitted and potentially spread by blood transfusions. Infections in pregnant women can spread to the baby [6]. Between January 1, 2018 and December 31, 2019, an average of 49 cases of malaria, 36 instances of dengue, and 10 cases of swine flu were reported in Gujarat, according to the statistics [7]. People need to be educated regarding Mosquito Borne diseases in order to opt preventive strategies for these conditions. A study conducted in Gujarat to study Awareness, precaution & Practice Regarding Mosquito Borne Diseases was concluded that the people were aware about Malaria and Dengue, but not about other mosquito borne diseases. This study recommended the need of creating awareness about other mosquito borne diseases [8].

## Methodology:

A comparative descriptive survey research design was used to complete the study. A sample is a subset of the population that has been chosen for observation and analysis. The process of picking a subset of the population to represent the complete population is known as sampling. Using a non probability convenient sampling method, a sample of 300 adult people (Rural 150+Urban 150) was selected from Mahesana district of North Gujarat. The rationale for selecting these areas was for feasibility of conducting research, easy access to subjects, availability of adequate samples, administrative approval and expectation of cooperation for conducting study. Structured Awareness Questionnaire & Checklist of precautions were used for data collection. Section-A consists of Demographic variables. I.e. Age, Gender, Education, Type of work, area of living, Sources of information, Type of house, previous history of mosquito bore disease, Uses of available of resources. In Section B Adults residing in chosen urban and rural areas were asked to fill out a structured questionnaire about their knowledge of mosquito-borne diseases. Section- C consists of checklist of possible precautionary measures against mosquito borne disease among adults living in selected urban and rural areas. Investigator divided awareness scores as following and considered samples awareness level accordingly. Good awareness level, awareness score between 14-20 out of 20, Average awareness level, awareness score between 7-13 out of 20 and Poor awareness level, awareness score between 0-6 out of 20. The investigator planned to analyze data by using descriptive and inferential Statistics. All the data has been analyzing by using frequency distribution, percentage and was presented in the form of the tables and graphs. The Correlation between Awareness and Precaution was shown by using Karl Pearson Correlation Coefficient formula refers to a process for establishing whether there any relationship exist between two variables or not. Chi Square test has been used to find association between selected demographic variables and Awareness as well as Precaution. A comparative descriptive survey approach was used in this study to assess the Awareness and Precaution on selected Mosquito Borne Disease among Adults in selected urban and rural areas of Mahesana district, Gujarat stare. The present study was conducted in selected urban and rural areas. The samples were collected using purposive sampling method. The samples population consists of 300 Adults For collection of data. Questionnaire were prepared and validated by experts. Pilot study was conducted in urban area on date of 1st march, 2022 and rural area on date of 7th March, 2022. Before starting the final data collection formal permission was obtained from concerned authorities. Final data collection was done from data 1st April, 2022 to 7th April, 2022. Investigator did not find any difficulty during data collection and finished within time limit.

## Results:

The demographic variables of the samples revealed that majority of samples were from age group 26-33 years (40.7%) in urban areas and (34.0%) in rural areas, majority of the samples were from male 89 (59.3%) in urban areas and 91 (60.7%) in rural areas, majority of samples were from higher secondary education 49 (32.7%) in urban areas and secondary education 55 (36.7%) in rural areas, majority of the samples were from private job 77 (51.3%) in urban areas and unemployed 62 (41.3%) in rural areas, majority of samples were from health worker 62 (41.3%) in urban areas and 56 (37.3%) in rural areas, majority of the samples semi pakka 69 (46 %) in urban areas and 71 (47. 3%) in rural areas, majority of the samples about not previous family history on mosquito borne disease 78 (52%) in urban areas and 120 (80%) in rural areas, majority of samples about not use of available of government services against mosquito borne disease 100 (66.7%) in urban areas and 116 (77.3%) in rural areas.

Figure 1(see PDF) shows that, in finding of the awareness of samples on selected mosquito borne disease, among 300 samples majority of samples had average (47.3 %) and poor (16%) awareness and (36.7%) had good awareness in urban areas and majority of the samples had average (40.67%) and poor (28%) awareness and (31.33%) had good awareness in rural areas.

In the precaution section, Table 1(see PDF) shows that among urban population, 67.3% were using Creams & Liquids and 22% were using Bed Net, 5.33 % were using Coil, 3.33 %were using Natural Remedies, 2% were using No precaution. Among rural population, 10% were using Creams & Liquids and 68.6% was using Bed Net, 3.33 % were using Coil, 12.6% were using Natural Remedies, 5.3% were using No precaution.

Five questions were used to assess MBD precautions, as indicated in Table 2(see PDF). Each question included a yes or no response option. YES received a score of 1, while NO received a score of 0, with a score range of maximum 5 to minimum 0. The scale rated practice as good if it received a score of 2 or above and poor if it received a score of 1 or 0.In urban areas Majority of the samples were cleaning the house daily (88%), Cleaning the surroundings weekly (57.3%), Cleaning water tank weekly (65.3%), Avoids stagnation of water (82%) and 98% people were using any one of the household methods. In rural areas majority of the samples were cleaning the house daily (93.3%), Cleaning water tank weekly (54.6%), Avoids stagnation of water (50.6%) and 94.6% people were using any one of the household methods. Cleaning the surroundings weekly was not done by majority (62.6%) in rural areas. In the association between awareness and selected demographic variables, the findings revealed that was significant association between awareness and Age, Gender, Education, Occupation, Type of work, Sources of Information, Type of house and previous family history on mosquito borne disease among adults at 0.05 significance. While there was no any association between awareness and Available Government services against mosquito borne disease. In the association between precaution and selected demographic variables, the findings revealed that there was no any association between precaution and Age, Gender, Education, Occupation, Type of work, Sources of information, Type of House, Previous family history of mosquito borne disease and Availability of Government services against mosquito borne disease. Table 3(see PDF) shows that in the area of introduction to MBD, urban population acquired a mean score of 52.38 % and rural population had 48.86%. In the Sign & symptoms area urban population had a mean score of 43.56% and rural population had 37.83%. Regarding Prevention of MBD, the urban population's mean score was 67.89% and rural population's was 60.83%. Regarding the Programs on prevention of MBD, urban people had mean awareness score of 47.67% and rural people had 28%. Figure 2(see PDF) shows that in the finding the correlation between awareness and precaution of samples on selected mosquito borne disease, there was moderate positive correlation between awareness and precaution calculated by Karl Pearson Correlation Coefficient formula which was 0.69 at 0.05 level of significant. This indicated that if awareness increase the precaution of samples will improve.

## Discussion:

From the above findings it was considered that the adults had average awareness on selected mosquito borne disease and majority sample were using precaution on selected mosquito diseases. The awareness and precaution are significantly correlated. This implies that as the awareness of samples on selected mosquito borne disease increases, their precaution level also increase. The study findings revealed that among the urban adults having more awareness than rural adults. A similar study conducted at New Delhi in 2020, also revealed that people have moderate awareness on various Mosquito borne diseases. Majority of them were not using proper preventive measures [9]. Another study conducted on Community Perception Regarding Mosquito-borne Diseases in Karnataka State, concluded that large number of people had inadequate awareness regarding the cause and spread of mosquito borne diseases [10]. The majority of the participants in this study used one or more methods to protect themselves from mosquito-borne diseases. Another study on community awareness of mosquito's and associated themes in the residential areas of two Tanzanian cities (Dares Salaam and Tanga) found that inhabitants were quite knowledgeable about mosquitos. Almost everyone said they use some kind of home mosquito repellent for personal protection, and many said they spend a large percentage of their household money on it. Although the interests of the citizens and the health authorities are clearly not the same, mutual cooperation is necessary.

Campaigns targeting against malaria vectors should address the need for additional methods to suppress Culex mosquitos, such as those now being tested by the UMCP, in order to preserve community support. Mosquito breeding sites are not specifically related with trash and standing water of any kind, thus the community's efforts to reduce mosquito sources are often ineffective. Residents are unaware that some mosquitoes carry malaria and others produce nuisance mosquitoes. Environmental anti-mosquito strategies being advocated through health education and other forms of propaganda are similarly ineffective. Some are focused at key Culex breeding locations, while others are aimed at sites with little or no significance for mosquitoes of any kind [11]. The importance of awareness and precautions against Mosquito borne diseases is known. It suggested that Mosquito-borne infections have become increasingly widespread, according to the study, as previously geographically isolated diseases have expanded globally. Chikungunya, dengue fever, Japanese encephalitis, malaria, West Nile virus, yellow fever, and Zika are only a few of the viral diseases conveyed by mosquitoes. To make a swift and accurate diagnosis, you'll need a complete patient history, physical, and knowledge of diagnostic testing based on symptom duration. Because many of these diseases have supportive treatments, the focus is on limiting infection risk and dissemination [12]. The precaution taken by the urban and rural population was satisfactory in the present study. This is supported by another research study named Mosquito Attractants in the year 2018. It suggested that Insecticides and repellents are the mainstays of vector management and personal protection against anthropophilic mosquitoes. For decades, researchers have been looking for mosquito-attractive semiochemicals, and new compounds or odour blends are frequently offered as lures for odor-baited traps. A thorough and up-to-date assessment of all research that have looked at the attractiveness of volatiles to mosquito's, including individual chemical compounds, synthetic blends of compounds, and natural host or plant scents. A total of 388 articles were reviewed, and our findings revealed that there are 105 attractants (77 volatile chemicals, 17 biological smells, and 11 synthetic mixes) that have been proven to attract one or more mosquito species [13].

## Conclusions:

Data shows that people have average awareness on selected mosquito borne disease especially in Introduction, Sign and Symptoms, Prevention and Programs. Majority of the people were using one or another method of precautions against mosquito borne diseases. Data also shows that the urban adults are having more awareness than rural adults. Hence, there is a need for awareness and IEC to make the adults free from misconception related to selected mosquito borne disease.
